# ROS Chronicles in HIV Infection: Genesis of Oxidative Stress, Associated Pathologies, and Therapeutic Strategies

**DOI:** 10.3390/cimb46080523

**Published:** 2024-08-14

**Authors:** R Harshithkumar, Prachibahen Shah, Pratiksha Jadaun, Anupam Mukherjee

**Affiliations:** Division of Virology, ICMR-National Institute of Translational Virology and AIDS Research, Pune 411026, India; harshith8398@gmail.com (R.H.); prachishah1999j@gmail.com (P.S.)

**Keywords:** OS, ROS, HIV infection, antioxidant therapies, pathogenesis, antiretroviral therapy, HIV associated dementia, Tat

## Abstract

Reactive oxygen species (ROS) are widely regarded as signaling molecules and play essential roles in various cellular processes, but when present in excess, they can lead to oxidative stress (OS). Growing evidence suggests that the OS plays a critical role in the pathogenesis of HIV infection and is associated with several comorbidities in HIV-infected individuals. ROS, generated both naturally during mitochondrial oxidative metabolism and as a response to various cellular processes, can trigger host antiviral responses but can also promote viral replication. While the multifaceted roles of ROS in HIV pathophysiology clearly need more investigation, this review paper unravels the mechanisms of OS generation in the context of HIV infections, offering insights into HIV viral protein-mediated and antiretroviral therapy-generated OS. Though the viral protein Tat is significantly attributed to the endogenous cellular increase in ROS post HIV infection, this paper sums up the contribution of other viral proteins in HIV-mediated elicitation of ROS. Given the investigations recognizing the significant role of ROS in the onset and progression of diverse pathologies, the paper also explores the critical function of ROS in the mediation of an of array of pathologies associated with HIV infection and retroviral therapy. HIV patients are observed with disruption to the antioxidant defense system, the antioxidant therapy is gaining focus as a potential therapeutic intervention and is well discussed. While ROS play a significant role in the HIV scenario, further exploratory studies are imperative to identifying alternative therapeutic strategies that could mitigate the toxicities and pathologies associated with ART-induced OS.

## 1. Introduction

**Figure 1:**
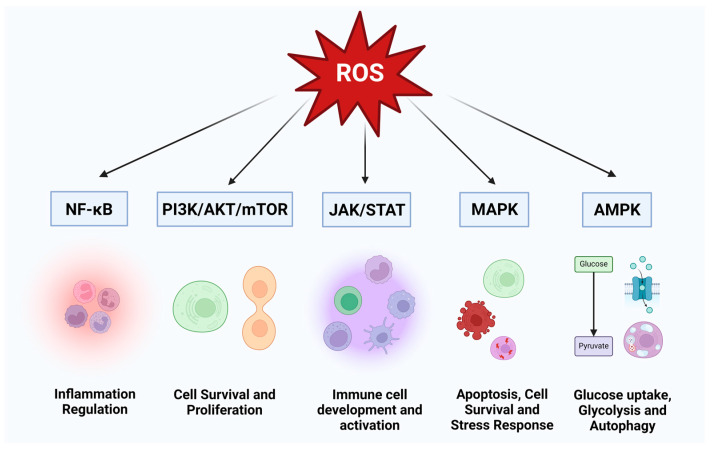
Regulatory Pathways Modulated by ROS and Their Impact on Cellular Functions. ROS-modulates several major cellular pathways including AMPK, JAK/STAT, NF-κB, PI3K/Akt/mTOR, and MAPK. These pathways regulate numerous cellular functions, such as energy homeostasis, antioxidant defenses, immune responses, inflammation, cell growth, survival, cell cycle regulation, and DNA repair. Variation of ROS levels in these pathways critically impact their cellular functions.

**Figure 2:**
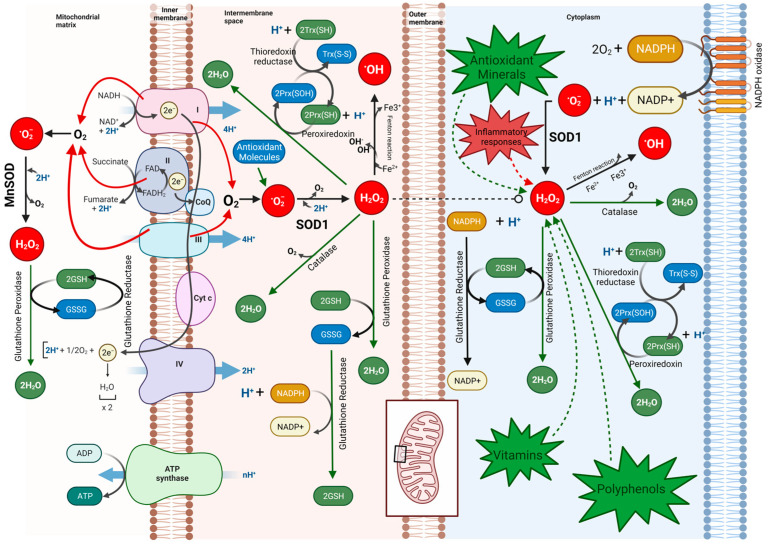
ROS Generation and Neutralization Pathways. Several pathways govern the generation and regulation of ROS within cellular environments. Notably, the mitochondrial electron transport chain is a significant source of ROS production, where escaping electrons engage with molecular oxygen, culminating in the formation of ROS species including superoxide anion and hydrogen peroxide. Additionally, the activation of NADPH oxidases (NOX) is shown, with these enzymes transferring electrons from NADPH to molecular oxygen, resulting in the generation of superoxide. Counteracting the potentially detrimental effects of ROS, the figure portrays key defense mechanisms, encompassing ROS-scavenging enzymes such as superoxide dismutases (SODs), catalase, and glutathione peroxidases. SODs convert superoxide into less reactive hydrogen peroxide, while catalase reduces hydrogen peroxide to water and molecular oxygen, and glutathione peroxidases eliminate hydrogen peroxide using the reducing power derived from glutathione. Further defenses involve peroxiredoxins, the thioredoxin (TRX) system, and the glutathione/glutaredoxin system, which collectively collaborate to uphold an equilibrium of ROS levels. Additionally, vitamins, polyphenols, antioxidant minerals, and the glutathione pathway neutralize the ROS, significantly maintaining cellular health.

The process of an HIV infection is characterized as an opportunistic and strategic manipulation of host cells, leading to a significant dysregulation of numerous host machineries [1,2]. Since the late 1980s, there has been speculation that reactive oxygen species or ROS contribute to the pathogenesis and progression of HIV-1 [3]. Being able to induce changes in the conformation of proteins, lipids, and glycans, the ROS can cause as much as a genetic mutation, impairing the critical functions of the system [4,5]. Many significant biological processes, such as immunomodulation, transcription, apoptosis, neuromodulation, cytokine signaling, ion transport, protein phosphorylation, hormone action, and broadly cell signaling, are influenced by ROS levels [6,7,8,9] (Figure 1). ROS are naturally produced during mitochondrial oxidative metabolism in response to various cellular processes and are also regarded as a second messenger in the MAP kinase cascade, controlling gene regulation and directly modulating redox-sensitive transcription factors [10] (Figure 2). The mitochondrial electron transport chain serves as the major site of ROS generation inside the cells where electrons escape their route and react with molecular oxygen, reducing it to form ROS such as superoxide anion (^•^O_2_^−^) and hydrogen peroxide (H_2_O_2_) [11,12]. Additionally, ROS can also be generated by the activation of NADPH oxidases (NOX), where NOX enzymes transfer electrons from NADPH to molecular oxygen, generating superoxide [13,14,15]. To prevent the detrimental effects of ROS and to keep a steady control of the production of ROS, cells have evolved a comprehensive array of defense mechanisms. These defense mechanisms primarily involve ROS-scavenging enzymes and antioxidants. The scavenging enzymes include superoxide dismutases (SODs), which convert superoxide (^•^O_2_^−^) to less reactive H_2_O_2_; catalase (CAT), which reduces H_2_O_2_ to water and molecular oxygen; and glutathione peroxidases (GPx), which eliminate H_2_O_2_ using reducing power derived from glutathione. Other defensive mechanisms encompass peroxiredoxins, the thioredoxin (TRX) system, and the glutathione/glutaredoxin system [16]. These intricate systems work in synchronization to maintain a balanced ROS level and ensure cellular homeostasis. However, the human immunodeficiency virus (HIV), known for its adaptability, has evolved the ability to interfere in ROS modulation and subvert host cellular pathways.

Although ROS are known to trigger the host antiviral response, many recent studies have revealed that an elevated intensity of ROS can also promote viral replication and propagation [17,18,19,20]. HIV induces a significant increase in ROS production, which can occur through direct interactions of HIV proteins in cellular pathways or as a consequence of immune responses such as elicited cytokine production, resulting in an imbalance of ROS homeostasis, referred to as Oxidative Stress (OS). The HIV infection is highly evidenced of having significantly elicited OS, benefitting the virus by exhausting the host antioxidant defense machinery, thereby weakening the immune system as immune cells require more antioxidants to maintain their function and integrity. The magnitude of ROS has been found to inversely correlate with the CD4+ cell count, which can be directly attributed to the impaired ROS defense system leading towards metabolic reprogramming [21,22,23,24,25]. Additionally, HIV-induced ROS activates the nuclear transcription factor NF-κB, which plays a crucial role in viral transcription and HIV latency, further benefiting the virus [26,27,28,29].

The complex interplay of ROS in HIV needs more understanding to elucidate the multifaceted roles of ROS in HIV infection. Given that ROS can exhibit both advantageous and detrimental effects on HIV, further research is imperative to unravel their intricate involvement. Moreover, it is evident that the generation of OS resulting from both antiretroviral therapy (ART) and HIV infection gives rise to numerous distressing pathological manifestations. The present review sheds light on the mechanisms underlying the induction of OS by the interaction of HIV and ART therapy, subsequently illustrating the associated pathologies and the potential intervention of antioxidant therapy in the context of HIV infections.

## 2. Molecular Interaction of HIV Proteins in ROS Generation

**Figure 3:**
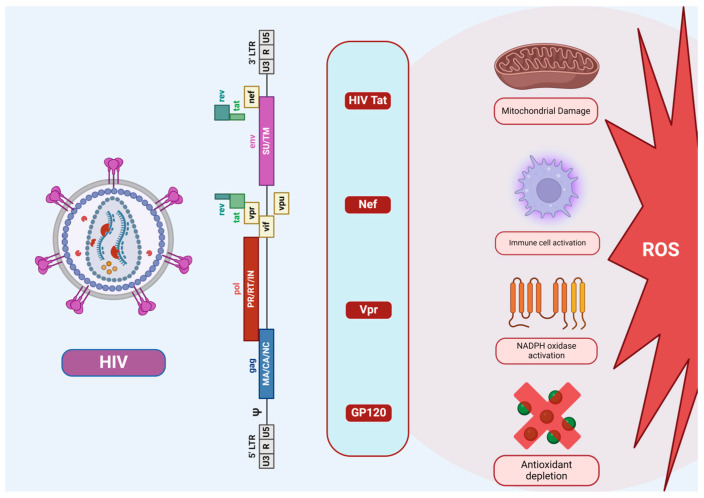
HIV-Induced Cellular Alterations Leading to ROS Production. The schematic illustrates the genomic structure of HIV and its impact on host cell oxidative stress. The key viral proteins (Tat, Nef, Vpr, GP120) contributes to cellular dysfunction, including mitochondrial damage, immune cell activation, NADPH oxidase activation, and antioxidant depletion which collectively lead to elevated ROS production resulting in OS within the host environment.

**Figure 4:**
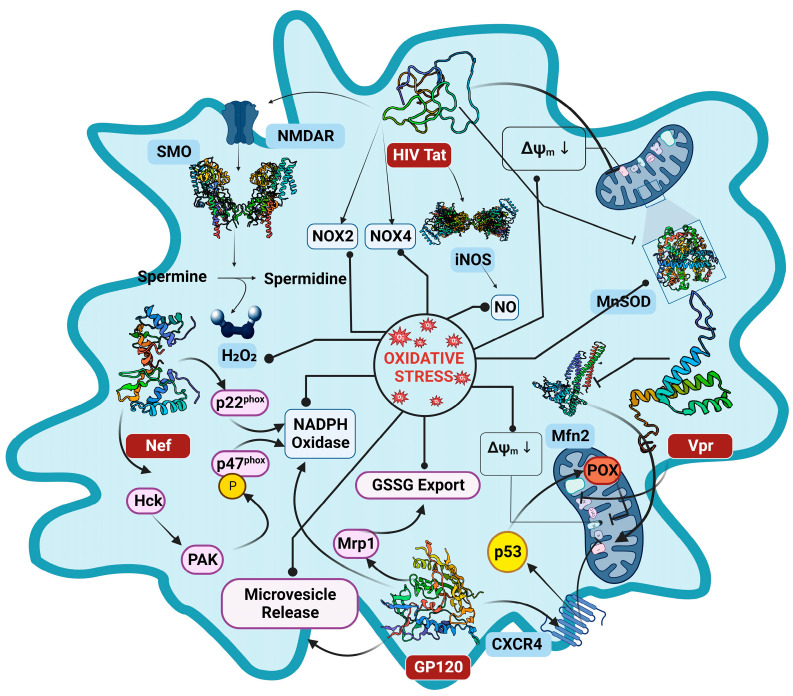
Molecular Mechanisms of Oxidative Stress Induction by HIV Proteins. The image illustrates the processes through which HIV proteins, including Tat, Nef, Vpr, and GP120, mediate the generation of ROS and subsequent OS. Each protein exerts distinct mechanisms leading to ROS production, contributing to the overall OS environment in HIV-infected cells. Tat translocates to the mitochondria, inducing mitochondrial transmembrane potential depolarization and suppressing antioxidant enzymes. GP120, akin to Tat, initiates mitochondrial transmembrane depolarization and affects the redox state of the cell. Nef upregulates superoxide anion release through NADPH oxidase complex modulation. Vpr induces OS by permeabilizing mitochondrial membranes and elevating reactive oxygen species. The image represents a comprehensive insight into the multifaceted role of these HIV proteins in OS induction.

The dynamics of ROS generation in the HIV infection scenario have long been attributed to HIV proteins. Numerous lines of research investigations evidenced the association of HIV and its viral proteins with significant ROS elevation, in both HIV infected patients and cell models [30,31,32]. The HIV infection deregulates the OS pathways by escalating ROS production and instigating mitochondrial dysfunction through viral proteins, leading to the induction of OS in the host environment [33,34] (Figure 3). It remains highly plausible that several viral proteins are involved to influence the OS paradigm, and the viral proteins Glycoprotein 120 (GP120), Trans-Activator of Transcription (Tat), Negative Regulatory Factor (Nef), Viral Protein R (Vpr), and Reverse Transcriptase (RT) each exhibit variegated roles and mechanisms in the context of OS pathways, that are discussed in detail in the following sections (Figure 4).

### 2.1. Tat (Trans-Activator of Transcription) Protein

Tat is one of the early HIV proteins that remarkably amplifies the viral transcription [35,36]. It recognizes the transactivation response element (TAR), a stem loop structure located at the 5′ end of the viral RNA transcript, and upon binding to it, Tat activates the transcription complex [37,38]. Tat also modulates many key pathways and has a magnitude of independent ways by which it can induce the production of ROS (Figure 4). The Tat protein is reported to translocate itself from the nucleus to the mitochondria of the host cell [39], where it interacts with the mitochondrial membrane to induce mtROS by eliciting the depolarization of mitochondrial transmembrane potential (MMP) [39,40]. In a different investigation conducted by H. Lecoeur et al., it was evident that Tat mediates the MMP through the dysregulation of anionic channels, leading to a surge in OS [39]. Furthermore, it is well reported that Tat can markedly repress the expression of manganese superoxide dismutase (MnSOD), a defense enzyme against OS [41]. The Tat protein can also induce OS by eliciting spermine oxidase (SMO) activity, by stimulating the N-methyl-D-aspartate receptor (NMDAR), activating the Nrf2/ARE Pathway leading to Glutathione exhaustion [31]. Another mechanism by which HIV-1 Tat augments the intracellular ROS concentration is by inducing the expression of two prominent NADPH oxidases (NOXs), NOX2 and NOX4 [42]. The NOX2 expression can be linked with the TNF-induced activation of NF-κB signaling pathway in an Akt-dependent manner [43], which can increase the formation of reactive oxygen intermediates by reducing mitochondrial superoxide dismutase (MnSOD) [44]. The reduction in MnSOD, together with the downregulation of Bcl-2 by the direct influence of Tat, can promote apoptosis as well [45,46]. The Tat-mediated OS is also associated with evoked expression of inducible nitric oxide synthase (iNOS), triggering the overproduction of NO radicals. The surplus of NO radicals reacts with superoxide anion to form a neurotoxin peroxynitrite and increases the glutamate release from astrocytes thus enhancing NMDA excitotoxicity [47]. The Tat protein is also established to trigger an increased production of inflammatory products, which may, in turn, cause an excess formation of ROS [48]. It is evident from the studies that the Tat protein dysregulates several pathways involving glutathione synthase (GSS), glutathione reductase (GR), and glutathione peroxidase (GPx) by increasing the GSSG/GSH ratio, which is reflective of a drastic escalation in OS [49].

### 2.2. The Envelope Protein GP120

The HIV envelope protein GP120 has been discovered to be essential in many aspects of HIV pathology, alongside facilitating the virus entry into the host cell. The induction of OS by GP120 was initially affirmed by exogenously exposing it to the cells, where the ROS production was significantly increased in various cells like astrocytes, microglia cells, T cells, and neurons, involving several mechanisms [32,50,51,52,53] (Figure 4). As reported by A. Shah et al., the GP120 mediates OS through the cytochrome P450 (CYP), NADPH oxidase (NOX) pathways, and the Fenton–Weiss–Haber reaction [32]. The GP120, similar to Tat protein, can induce mitochondrial transmembrane depolarization, triggering the production of ROS [54]. A study by Pandhare et al. confirmed that the GP120 treatment induced POX expression through p53, and the catalytic activity of proline oxidase (POX) in GP120 mediated OS [55]. The Multidrug resistance protein 1(Mrp1), known to efflux GSH and GSSG, is functionally regulated by GP120 protein. The Mrp1 expression is amplified by the GP120 [56], leading to the heightened export of GSSG, perturbing the redox state of the cell and eventually guiding the cell to apoptosis [57]. The HIV-1 GP120 can stimulate microvesicle release from endothelial cells, and these endothelial microvesicles (EMVs) confer pathological effects on endothelial cells by inducing OS [58]. The envelope protein may as well trigger OS in bystander CD4+ T cells, expediting uninfected CD4+ T cell death by apoptosis [59]. The GP120-mediated OS is also linked with cofilin–actin rod formation, which contributes to the early synaptic dysfunction observed in HIV-associated neurocognitive disorder (HAND) [50]. The GP120 is also shown to cause lipid peroxidation and the production of hydroxynonenal esters [60]. Much similar to the Tat protein, the envelope protein also downregulated the expression of antioxidant enzymes such as glutathione synthase, glutathione peroxidase, glutathione oxidoreductase, and increased the GSSG/GSH ratio [24].

### 2.3. Negative Regulatory Factor

The HIV-1 Negative Regulatory Factor (Nef) is a nonenzymatic accessory protein known to be vital in maintaining the persistence of HIV infection by enhancing viral replication and promoting immune evasion. The Nef protein exhibits significant upregulation during the early stage of infection but maintains its expression throughout all subsequent stages, interfering with diverse host signaling pathways [61,62]. Olivetta et al. investigated the effects of Nef on the NADPH oxidase complex in human macrophages and reported that the Nef regulated the (^•^O_2_^−^) release by modulating NADPH oxidase activity through the phosphorylation of the p47(phox) subunit of the NADPH oxidase complex by Nef-activated Src kinases and PI3K [63]. Nef was also found to associate with p22-phox, another subunit of NADPH oxidase complex, increasing the superoxide production [64]. Nef could also activate the NADPH oxidase indirectly, by triggering the Vav/Rac/p21-activated kinase (PAK) signaling pathway involved in the instigation of phagocyte NADPH oxidase [65]. Masanetz et al. investigated the sensitivity of astrocytes towards hydrogen peroxide and concluded that the Nef protein contributes to a decrease in the tolerance of astrocytes, leading to neuronal dysfunction and HIV-associated dementia (HAD) [66], while it is well reported that HAD patients show an elevated expression of Nef protein in the astrocytes [67].

### 2.4. Viral Protein R

The Viral Protein R is a 96 amino acid conserved protein that aids in the execution of multiple functions like nuclear import of the HIV-1 pre-integration complex, transactivation of the viral promoter, and facilitates the infection of nondividing cells [68,69]. The infected macrophages are demonstrated to release a substantial amount of Vpr, which could be detected in the sera and cerebrospinal fluid (CSF) of a large group of HIV-1-infected patients [70]. The Vpr is well attributed to instigating OS by the induction of mitochondrial membrane permeabilization (MMP) through direct interaction with the adenine nucleotide translocator (ANT) present in the inner membrane of mitochondria [71]. It is also previously shown that Vpr increases the permeability of mitochondrial outer membrane (MOM) and subsequent loss in membrane potential (MMP) by post-transcriptionally reducing the expression of mitofusin 2 (Mfn2) via VprBP–DDB1–CUL4A ubiquitin ligase complex [72]. Deshmane et al. revealed in their investigation that the Vpr can induce the production and accumulation of the ROS in the host environment, leading to the activation of the hypoxia inducible factor 1 alpha that further cascades the activation of the HIV-1 promotor [73]. The activity of Vpr is also confirmed in the *S. pombe* cellular model that the intervention modulates the oxidative status of host cells and thus facilitates the disease progression [74].

### 2.5. Reverse Transcriptase

Reverse transcriptase (RT) has more or less a conserved functional role of DNA polymerization in HIV pathology. Although the association of RT is least ascribed to OS generation, Isaguliants et al. first described the induction of OS by HIV-1 reverse transcriptase. The investigation involved the expression of HIV-1 RT in human embryonic kidney cells and demonstrated the production of the ROS using fluorescence-based probes [75]. The latter results were confirmed in a separate study by Bayurova et al. in cells expressing consensus reverse transcriptase of HIV-1 [76], but much research is needed to decipher the actual mechanism of ROS generation by RT.

## 3. HIV ART-Associated OS

Antiretroviral therapy is a successful treatment regimen to reduce viral load in HIV-infected patients. To date, great improvements in combination antiretroviral therapy (cART) have transformed HIV-1 infection from a deadly illness to a tolerable chronic condition [77,78]. However, numerous reports from preclinical and clinical studies show that all categories of anti-HIV drugs are capable of generating OS at varied levels. The effects of active antiretroviral combinations on OS were assessed in an investigation by Ngondi et al., and the study found an increase in lipid and protein oxidation and a decrease in the cellular antioxidants abundance [79]. These findings were supported by another independent investigation which demonstrated the damage to membrane polyunsaturated fatty acid by antiretroviral drugs, which further lead to the generation of more free radicals [80]. Also, the free radical production was well elicited due to decreased selenium levels in the patients receiving ART therapy [81]. The antiretroviral-drug-mediated OS is majorly attributed to the mitochondrial dysfunction [82]. The dysfunction arises as a consequence of inhibition of mitochondrial DNA replication, as well as oxidative phosphorylation, which is further linked with many HIV-associated pathologies [83]. Most nucleoside reverse transcriptase inhibitors (NRTIs) preferably target the mitochondrial DNA polymerase-gamma, and that explains the inhibition of mt-DNA replication and associated dysfunction [84].

AZT (zidovudine), an effective NRTI of HIV replication and the first FDA-approved antiretroviral drug for the treatment of AIDS, is very well known to induce OS in host cells by inhibiting mitochondrial DNA replication. Specifically, the active metabolite AZT-triphosphate altered the mitochondrial dynamics to an extent leading to cell death [85]. This drug-induced OS and subsequent toxicity formed one of the reasons for its non-recommended status in HIV-1 therapy. Lamivudine, abacavir, and tenofovir are all extensively proven to interfere with mitochondrial ROS balance and provoke OS generation. The administration of lamivudine and tenofovir were clearly shown to induce OS and related toxicities in the *Drosophila melanogaster* model as well [86]. Tenofovir administration in adult Wistar rats reduced the glutathione by 50%, along with a reduction in the activity of superoxide dismutase, glutathione peroxidase, and glutathione reductase by 57%, 45%, and 150%, respectively, relating the tenofovir-induced OS particularly to the depletion of the antioxidant system [87]. Abacavir and didanosine, the other commonly prescribed purine analogues, are well linked with mitochondrial dysfunction, prominently leading to OS-induced hepatotoxicity [88].

The similar scenario would be in the case of most commonly administrated Non-Nucleoside Reverse Transcriptase Inhibitors (NNRTIs). One of the widely prescribed NNRTI, efavirenz, is reported to elicit OS by directly affecting the mitochondrial function by decreasing MMP and by increasing mitochondrial superoxide production [89,90]. Rilpivirine, a second-generation NNRTI, also disrupts the membrane potential of mitochondria and increases ROS production. Additionally, the in silico docking analysis by Maandi et al. predicted a possible inhibition of mitochondrial ATP synthase by rilpivirine, supporting the elicited production of ROS upon the drug administration [91].

Protease inhibitor (PI) drugs are one of the crucial components of highly active antiretroviral therapy and are almost always prescribed in combination with other antiretroviral drugs. The generation of OS by PI is also quite reported. In a study by S. Chandra et al., it was documented that Nelfinavir, a protease inhibitor employed in ART therapy, significantly increased the ROS by suppressing cytosolic SOD levels [92]. Ritonavir is another PI that is continuously used since its approval by the U.S. FDA in 1996. The drug poses a high inhibitory activity against host hepatic cytochrome P450 3A4 [93], and hence it is currently administrated in lower doses as a pharmacokinetic enhancer for co-administered protease inhibitors [94]. Xi Chen and I. Tong Mak revealed in their study that a clinically significant dose of Ritonavir (15 µM) was able to increase superoxide production in bovine endothelial cells by 41% [95]. A protease inhibitor developed from Ritonavir, and which shares structural similarities with Ritonavir, Lopinavir, can also induce OS [96,97].

Contrary to the above protease inhibitors, Atazanavir is attributed to a lower degree of OS. OS markers were observed in HIV-infected patients on efavirenz or atazanavir/ritonavir administration in a study. The outcome of the investigation documented that OS was lower in ATV/ritonavir regimens, compared to efavirenz, relating it to the increased unconjugated bilirubin upon ATV administration [98]. Similarly, another most clinically used PI, darunavir, was found to not be actively contributing to the production of ROS, delivering a potential advantage of lower OS over other PIs in the treatment regimen [97].

HIV Integrase Strand Transfer Inhibitors (INSTIs) are the recent compounds employed in antiretroviral therapy. The first INSTI to get FDA approval for clinical use was Raltegravir (RAL) in the year 2007, followed by another potent INSTI Dolutegravir (DTG) in 2013. In light of the remarkable efficacy of INSTIs in reducing viral load, they are now being highly recommended in HIV treatment regimens worldwide, as a first-line treatment in most countries for people living with HIV [99]. The information on OS generation by INSTIs is comparatively less and not very well explored. But a study by T. Latronico et al. evaluated the effect of RAL in cultured primary astrocytes and showed that RAL can induce OS [100]. Similar results were documented in another study led by J. Gorwood et al., where they observed the ROS production by RAL and DTG in proliferating adipose stem cells (ASC) [101]. The group also reported increased mitochondrial dysfunction characterized by decreased membrane potential and increased mitochondrial mass in proliferating ASCs and adipocytes [101]. While DTG is the preferred first-line drug, its potential to induce oxidative stress raises concerns about long-term metabolic effects in patients. The heightened ROS production post DTG administration was also confirmed by significantly more DCFDA fluorescence in the drug-exposed erythrocytes [102]. This may be linked to the fact that DTG can trigger suicidal death in erythrocytes as characterized by cell shrinkage. The brains of the adult mice are also reported having OS upon DTG treatment, suggesting damage to the CNS by the drug [103]. The implication of DTG on longevity is a focal point in the current situation. A study conducted by Kuo SH and team explored to determine the DTG-induced growth and lifespan effects in C. elegans models. The results demonstrated that the DTG treatment enhanced the intracellular ROS accumulation in C. elegans in a dose-dependent way and is presumably the cause of the reduced lifespan since treatment with NAC significantly extended the nematodes’ lifespan [104]. While DTG treatment is known to result in transient calcium efflux and aberrant mitochondrial membrane potential, leading to significant ROS production, a recent investigation reported the generation of endoplasmic reticulum stress by DTG that eventually disrupted the murine blood–brain barrier [105]. Drug nano-formulation approaches are being investigated for the attenuation of OS caused by DTG, which have shown promising results even in reducing brain OS [103,106].

## 4. ROS-Associated Pathologies

**Figure 5:**
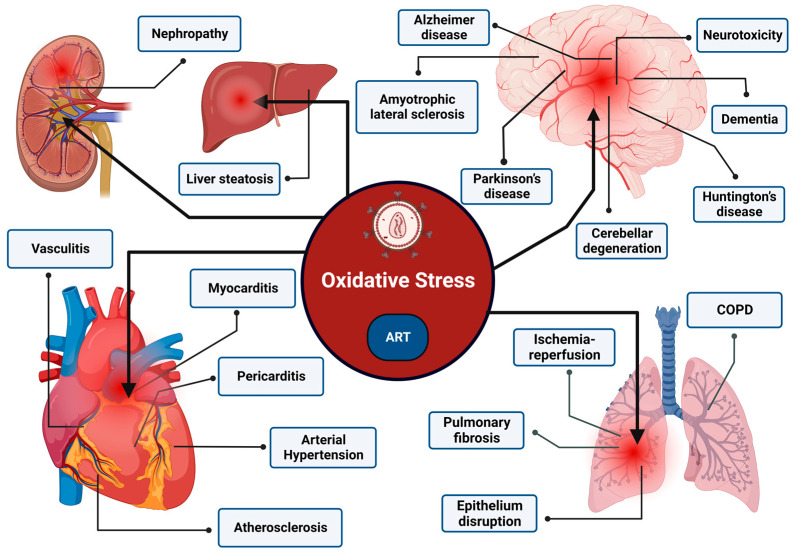
Illustration of ROS-Mediated Pathologies in HIV Infection and ART. The schematic representation depicts the multi-organ impact of OS induced by the interplay of HIV infection and antiretroviral therapy (ART). The brain exhibits pathologies such as neurotoxicity, dementia, Alzheimer’s disease, amyotrophic lateral sclerosis, Parkinson’s disease, cerebellar degeneration, and Huntington’s disease, all influenced by OS. Additionally, the liver is affected by steatosis, the kidney by nephropathy, and the heart by vasculitis, myocarditis, pericarditis, arterial hypertension, and atherosclerosis, all attributed to OS. In the lungs, HIV infection and ART contribute to chronic obstructive pulmonary disease (COPD), ischemia-reperfusion injury, pulmonary fibrosis, and epithelial disruption, with OS as a key mediator. Understanding these intricate relationships is vital for managing and mitigating the diverse pathologies associated with HIV infection and its treatment.

The OS induced by the interplay of HIV infection and antiretroviral therapy (ART) can exert a significant impact on the onset and progression of diverse pathologies (Figure 5). This can have broad-ranging ramifications, exerting effects on multiple organ systems. The most major of all, HIV can enter the Central Nervous System (CNS) and cause a persistent infection, leading to a spectrum of neurocognitive dysfunction referred to as HAND (HIV-associated neurocognitive disorder) [107]. Y. Wang reported, in one of their meta-analysis, a high prevalence (42.6%) of HIV-associated neurocognitive disorders (HAND) in the HIV-infected population, suggesting an excessive burden of neuro-pathologies [108]. Evidence advocates that the chronic inflammation leading to the neurodegeneration observed in HIV infection is predominantly associated with ROS hyperproduction. A study by J. Louboutin et al. substantially demonstrated the mediation of OS in generating neuroinflammation when Rat caudate-putamens were administrated with HIV Tat [109]. The intervention of OS is also reported in facilitating neuronal apoptosis. The investigation by B. Shi et al. examined if the exposure of human neurons to HIV-1 Tat and TNF-alpha can instigate neuronal apoptosis, and they clearly identified that, through increasing the OS, both Tat and TNF-alpha can cause neurotoxicity thus leading to neuronal apoptosis in the brain of AIDS patients [110]. The clear relationship of OS in HAND is exemplified where reduced volumes of the hippocampus and subcortical gray matter correlated with increased oxidative mitochondrial DNA damage in peripheral blood mononuclear cells in chronically HIV-infected patients [111]. In addition to other contributing factors, lipid peroxidation also plays a role in the development of neurological disorders by contributing to neurotoxicity and subsequent pathology.

Dementia is one of the ugly repercussions of HIV sicario, characterized by the decline in cognitive functioning. HIV patients often present with complaints such as a lack of concentration, memory deficit, reduced attention, and the gradual loss of motor skills, all of which are indicative of dementia [112]. Alzheimer’s disease (AD) is the most common cause of dementia. Studies have reported that CSF features of individuals with HAND share similarities with the clinical and biological characteristics of AD patients with no HIV infection. Phosphorylated tau (P-tau) in CSF, which is an established biomarker that is seemingly specific for AD, has been reported to be found in the neurofibrillary tangles (NFTs) occurring in people suffering from HIV [113]. A study by Young-Eun Cho et al., designed to investigate the mechanisms of increased neurodegeneration in HIV-1 infection, demonstrated the neural cell death and degeneration upon elicited reactive nitrogen species (RNS) nitroxidative stress and subsequent Tau phosphorylation in HIV-1 transgenic rats [114]. Alongside RNS, there is clear evidence that ROS can directly promote tau modifications [115]. Studies have also shown that OS can promote Amyloid-Beta (Aβ) synthesis by diminishing the α-secretase activity and increasing the β and γ-secretase activity, leading to AD [116]. Aligning with it, the SOD1 deficiency in the amyloid precursor protein-overexpressing mouse model (AD mouse, Tg2576) is also found to accelerate the Aβ oligomerization and memory impairment, depicting the critical role of ROS homeostasis in AD progression [117].

Another ailment that exhibits pathological hallmarks akin to those of AD is Parkinson’s disease (PD). It is observed that around 5% of people living with human immunodeficiency virus (HIV) infections develop PD-like features [118]. The pathogenesis of PD is characterized by the gradual deterioration of dopaminergic neurons (DAns), which is predominantly attributed to the pivotal role played by mitochondrial dysfunction and augmented OS [119]. Accumulation of α-synuclein (α-syn) cytoplasmic inclusions, termed Lewy bodies, is another defining feature of PD where mounting evidence supports that the Lewy body formation is intimately linked to elicited oxidative and nitrosative stress [120,121,122]. Furthermore, the Lewy body in its intricate structure, exhibits a significant accumulation of oxidized proteins and lipid peroxidation-derived products [123]. These findings provide compelling evidence that the OS elicited by HIV infection may contribute to the pathogenesis of PD.

In conjunction with AD and PD, the OS engendered by both HIV infection and ART therapy can be speculated to exert an influence on the advancement of neurological conditions like amyotrophic lateral sclerosis (ALS), Huntington’s disease (HD), and cerebellar degradation [124]. An investigation estimated the incidence of ALS in the HIV populace and reported that 3.5 cases per 1000 patients were identified with ALS [125], juxtaposed to 4 to 6 cases per 100,000 individuals in the general population [126]. Also, it is a clear fact that OS mediates the ASL progression as evidenced by the presence of markers of lipid peroxidation, including 4-hydroxynonenal (4-HNE) and malondialdehyde (MDA) in the spinal fluid from ASL patients [127]. Hence, the heightened incidence of ALS cases observed in HIV-infected individuals can be correlated to the elicited OS that progresses the ASL [128]. But in the HD sicario, despite the lack of definitive evidence linking the progression of it in HIV-infected individuals, the well-established role of OS in HD pathogenesis suggests that HIV-induced OS may contribute to the advancement of HD. Thus, it is reasonable to speculate that the co-occurrence of HIV and HD may accelerate disease progression, necessitating further investigation into this complex interaction [129].

The OS provoked by ART also bears a commensurate impact on the propagation and exacerbation of myriad pathologies. The investigation by a group of researchers led by Cagla Akay et al. reported the potential toxicity of antiretroviral drugs in the central nervous system (CNS) by evincing the neurotoxicity of ART drugs in the CNS of both pigtail macaques and rats. In the study, the drug’s proclivity to stimulate the accumulation of ROS, thereby culminating in the induction of neuronal damage was well demonstrated, and it proffered substantive evidence that ART drugs can have a role in progressing ROS-associated neural damage [130]. Although, the reports greatly regard the reduction in HIV-associated dementia post antiretroviral therapy [131,132], a study by Ellen C. Caniglia et al. reported that the antiretroviral therapy regimen with a high CNS Penetration Effectiveness (CPE) score greatly increased the risk of HIV dementia [133].

The OS by antiretroviral drugs is also known to mediate pathologies associated with cardiotoxicity, including dilated cardiomyopathy, myocarditis, pericardial effusion, endocarditis, and pulmonary hypertension [134]. The intervention of NRTI is capable of inducing oxidative damage to cardiac mitochondria. During one study by José García de la Asunción et al., it was observed that the cardiac mitochondrial DNA (mtDNA) of mice, which had been subjected to AZT treatment, had an increase of over 120% in the levels of oxo-dG (8-oxo-7,8-dihydro-2′-deoxyguanosine), a biomarker that indicates damage caused by OS to DNA when compared to the untreated control group. Furthermore, the AZT treatment also led to an elevation in mitochondrial lipid peroxidation and oxidation of mitochondrial glutathione [135]. Alongside this, myocarditis and dilated cardiomyopathy represent two frequently encountered myocardial disorders in individuals with HIV infection, which are often characterized by a continuum of disease progression. Notably, HIV infection itself is recognized as a prominent etiological factor in the development of dilated cardiomyopathy, exhibiting a prevalence rate of 3.6% amongst patients diagnosed with this cardiac condition [136]. The presence of both the HIV infection and dilated cardiomyopathy in afflicted individuals confers a significantly elevated risk of mortality, with four times more mortality observed relative to patients with isolated idiopathic dilated cardiomyopathy devoid of HIV infection [137]. The impact of NRTIs on cardiac pathologies was evaluated in a study by A. Pugliese et al., where they reported that around 51.8% of patients treated with NRTI developed cardiac involvement [138]. This outcome could be attributed to the mitochondrial damage caused by the NRTI drugs, contributing to cardiomyopathy [139].

The HIV-infected patient also experiences a significant burden of morbidity related to liver conditions. Antiretroviral therapy is regarded as a risk factor for non-alcoholic fatty liver disease (NAFLD) progression, mainly due to the associated mitochondrial dysfunction with the use of certain antiretroviral drugs. The mitochondrial dysfunction also seems to induce adipocyte apoptosis, leading to peripheral lipodystrophy [140]. The death of adipocyte results in loss of fat tissue in the peripheral areas of the body, leading to visible changes in body fat distribution. Directly, the HIV glycoprotein GP120 receptor can instigate the hepatic stellate cells (HSCs) to activate metabolic pathways that produce ROS [141], leading to ROS-mediated liver pathologies like NAFLD, fibrosis, and hepatic encephalopathy [142]. Together with that, reports have also claimed the deterioration of kidney function in HIV-infected individuals, termed HIV-associated nephropathy (HIVAN). The significance of OS in the onset and progression of HIVAN is being increasingly recognized [143,144,145]. Evidence also reported the apoptotic phenotype in tubular cells induced by the HIV infection [146], where OS is documented to be involved in the HIV-instigated kidney cell apoptosis [147,148]. Nephrotoxicity is strongly associated with increased OS due to antiretroviral therapy. The ROS due to the antiretroviral drug is reported to induce gene activation of oxidative stress responsive maladaptive pathways, thereby promoting apoptosis in glomerular cells and leading to nephropathy [149]. Moreover, adefovir and tenofovir are related to tubular toxicity, while crystal nephropathy and nephrolithiasis have been reported with indinavir, and acute interstitial nephritis has been reported in indinavir and atazanavir administered individuals. Notably, OS has been reported to play a key role in all of these medical conditions [150,151,152,153].

Chronic obstructive pulmonary disease (COPD) is a lung disease, causing restricted airflow and breathing problems. Growing evidence suggests that HIV is associated with COPD and a decline in lung function [154]. Although high rates of smoking and other confounding factors in HIV-infected populations complicate discerning HIV as a risk factor for COPD, many reports have proposed HIV as an independent risk factor [155,156]. The HIV infection is also associated with pulmonary fibrosis, emphysema, and increased susceptibility to infections [157], and the pathophysiological basis underlying the progression of these pulmonary disorders is thought to involve OS-mediated mechanisms [158]. The occurrence of OS within the pulmonary microenvironment gives rise to a reduction in the expression of tight junction receptors, which results in the disruption of the epithelium and consequently, enhances the vulnerability of the lungs to microbial infections [159]. Further, in HIV infection, oxidative stress has been identified as a significant factor contributing to adverse outcomes in HIV-associated tuberculosis (TB). Studies suggest that the oxidative stress associated with HIV infection may exacerbate the effects of TB by promoting the generation of metabolically dormant mycobacterial-persisters and through other mechanisms [160]. This interplay can lead to more severe disease progression and complicate treatment efforts [161].

## 5. Antioxidants in HIV Therapy

Antioxidants are associated with the modulation of numerous biochemical and physiological functions in the body. Several investigations suggesting the role of elicited OS generated by both HIV infection and ART in HIV progression and related pathology, along with decreased cellular antioxidant levels, signify the need for antioxidant supplements in the treatment regimen. The previous investigations, as discussed earlier, clearly reported the reduced tissue concentration of antioxidants like glutathione, ascorbic acid, tocopherols, carotenoids, and selenium in HIV-infected patients. Further, the antioxidant enzymes including SOD and GPx were also documented to be reduced during HIV infection [162,163]. Although essential antioxidants are produced endogenously to neutralize OS, HIV infection and ART add up to the increased cell starvation for antioxidants. Hence, the augmentation of antioxidant levels in the cell instinctively occurs to hinder OS associated pathologies. Few investigations depicted that the dietary intake of antioxidants can slow the progression of HIV. A clinical trial was conducted by T. Stewart et al. on adults under a stable ART to decipher the effectiveness of antioxidants in reducing oxidative damage. The intervention included multivitamins, alpha-lipoic acid, N-acetylcysteine, and acetyl-L-carnitine, and subjects were then assessed for the mitochondrial disfunction markers. The study concluded that the antioxidant supplements effectively prevented mitochondrial stress while improving the immune status [164]. Moreover, the administration of antioxidants can also inhibit the ROS-dependent signaling pathways at the cellular level, in turn inhibiting ROS activated autophagy [165], and they could therefore find a place in antiretroviral therapies as a strategy to extend the life span of AIDS patients.

Vitamins (Vit) play a crucial role in maintaining redox balance, yet their effects on OS in HIV patients remain understudied. HIV-positive individuals often exhibit lower levels of vit A, C, and E. Jaruga et al. demonstrated that supplementation with these vitamins in asymptomatic HIV patients restored antioxidant enzyme activity and reduced DNA oxidation [166]. Kpewou et al.’s cross-sectional study of 103 HIV-positive patients on antiretroviral therapy revealed significantly reduced serum vit E levels, highlighting the persistent deficiency despite treatment [167]. Further research showed that exogenous supplementation of vit E and C effectively reduced OS, associated lipid peroxidation, and viral load to some extent [168]. Complementing these findings, Spada et al. observed that vit E supplementation enhanced the effectiveness of antiretroviral therapy, suggesting a potential synergistic effect [169]. But in the case of vit A, HIV-infected populations showed reduced levels, but studies on its supplementation yielded conflicting results. Some reported benefits, while others indicated potential OS induction in animal models [170,171,172,173].

The increased ROS index is greatly tagged with depleted CD4+ cell counts [21,174], but the oral supplementation of micronutrients can increase the CD4 count in HIV-infected individuals on highly active antiretroviral therapy [175]. The supplementation of selenium in ART-naive patients also proved to significantly reduce the CD4 cell decline [176]. Likewise, the supplementation of N-Acetylcysteine (NAC), the acetylated precursor of reduced glutathione (GSH), also enhanced the CD4 cell count in HIV-infected individuals. In conjunction, N-Acetylcysteine administration also showed positive results in reducing the OS caused by combined ART in rat primary microglial cells [177]. V. Visalli et al. investigated the effect of NAC on GP120-mediated OS in human cultured astroglial cells and concluded that NAC countered the lipid peroxidation caused by the GP120 [178]. A similar investigation by T. O. Price et al. attempted to determine the effects of N-acetylcysteine amide (NACA) on GP120 and Tat-induced OS. The results were in alliance, showing a significant increase in the status of intracellular GSH, CAT activity, and GR activity, proving that NACA can reverse GP120- and Tat-induced OS in immortalized endothelial cells [179]. Nanozymes are regarded as the low-cost alternatives of natural enzymes. Vanadium pentoxide (V_2_O_5_), a nanosheet that functionally mimics natural glutathione peroxidase activity was reported to be successful in ROS neutralization and subsequent HIV-1 suppression. The combination of V_2_O_5_ nanosheets with a pharmacological inhibitor of NF-κB also inhibited the activation of ART-suppressed HIV-infected primary CD4^+^ T cells [180]. Recent studies have investigated the new-generation antioxidant molecules against HIV infection. Mitoquinone mesylate (MitoQ) is a synthetic analogue of coenzyme Q10 that has antioxidant properties and is approved for human use. In a study where MitoQ was administered alongside antiretroviral therapy, it showed promise in reducing mitochondrial dysfunction in multiple organs during chronic HIV infection, as demonstrated in a humanized mouse model [181].

Phytochemicals are a rich source of metabolites that possess antioxidant properties. Polyphenols like bioflavonoids and tannins are natural antioxidants found in plants [182,183,184]. A team of investigators led by J. Jaiswal working on the biochemical characterization of chemical components from *Parthenium hysterophorus* and its therapeutic potential against HIV-1 RT, evaluated the total antioxidant capacity of the plant and recorded that the *P. hysterophorus* extracts exhibit the presence of many phytochemicals with strong antioxidant properties [185]. In a similar study, the extracts from the rhizome of the plant *Curcuma aeruginosa* Roxb. also showed significant antioxidant activity, mostly attributable to the total phenolic content of the extract [186]. The extracts of *Satureja spicigera* (aqueous) and *Hoodia gordonii* also showed good antioxidant properties alongside inhibiting the HIV-1-RT activity, as reported in two distinctly conducted studies [187]. Likewise, the leaf extract of *Carpobrotus edulis*, an edible plant employed in the traditional medicine of South Africa, demonstrated inhibitory effects on free radicals, as evidenced by its hydrogen peroxide scavenging activity [188]. Extracts from another two plants of ancient medicine, *Gasteria bicolor* and *Pittosporum viridiflorum*, also showed in vitro antioxidant properties by capably scavenging the free radicals. In addition, a study from our research group identified the potent antioxidant activity of *Carica papaya* Linn and *Psidium guajava* leaves’ extract together with antiviral properties in an HIV infection environment [189]. A phytoestrogen from red grape called Resveratrol was demonstrated to reduce the viral load of HIV-1 infected macrophages while showing antioxidant properties [190]. Apart from plants, marine algae have also been identified as a valuable source of bioactive compounds, which may have the ability to act as antioxidants against HIV and ART-generated OS. The extracts derived from marine brown algae *Padina tetrastromatica* were shown to have significant antioxidant activity coupled with immune stimulatory functions [191]. Furthermore, our group endeavored to investigate the antiviral and antioxidant properties of a phycobiliprotein, C-Phycocyanin (C-PC). The investigation effectively elucidated the antioxidant potential of C-PC, where the C-PC ameliorated the ROS production in the HIV-infected cells, apart from inhibiting the virus replication [192].

## 6. Conclusions

**Figure 6:**
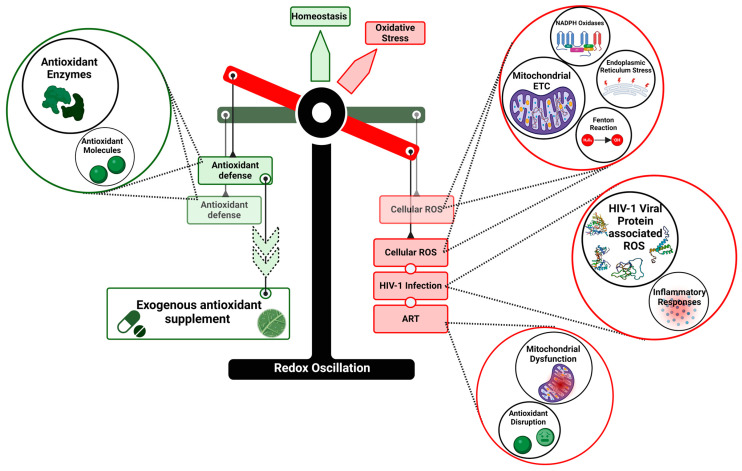
Redox Oscillation in HIV infection. Illustration depicting the alterations in redox balance by HIV-1 infection, leading to a deficit in antioxidant defense mechanisms that maintain redox homeostasis, and consequently OS generation.

The cellular redox balance is a consequence of dynamic counteracting interactions, and any disturbance in it would lead to the deficit of antioxidant defense, prompting OS (Figure 6). The relationship between HIV infection, ART, and OS presents a complex hurdle in the management of HIV/AIDS. The dual role of ROS in HIV infection presents a significant challenge. On the one hand, ROS can trigger host antiviral responses, potentially beneficial in controlling viral replication. On the other hand, excessive ROS production clearly contributes to cellular damage and various HIV-associated pathologies. This duality highlights the need for a more refined approach to managing OS in HIV patients.

The contribution of individual HIV proteins to OS, particularly Tat and GP120, is well-documented. However, the interaction between these proteins and their cumulative effect on ROS production is not fully understood. Future studies should adopt a more holistic approach, examining how these proteins work in concert to alter the cellular redox state. ART-induced OS reveals a concerning trend. While newer antiretroviral drugs have improved efficacy and reduced side effects, many still contribute significantly to OS. This is particularly evident with some integrase inhibitors, which were initially thought to have a better safety profile. Although ART has significantly improved the life prospects for patients living with HIV, the high prevalence of HAND and other HIV-associated pathologies despite viral suppression suggests that OS by ART drugs may be the key drivers of this impairment. The apprehensions show the need of longitudinal studies to address how OS may affect longevity. Even the current evidence base of antioxidant therapies in HIV management is a bit inconsistent, but the potential seems promising. Our understanding of the role of ROS in HIV infection has grown substantially yet many questions still remain unanswered. The concrete role of ROS in the immune modulation of HIV infection, latency, and in co-infections, are far from being deciphered and the answers to these may open up a way for the superior treatment of HIV/AIDS.
